# Citric Acid Confers Broad Antibiotic Tolerance through Alteration of Bacterial Metabolism and Oxidative Stress

**DOI:** 10.3390/ijms24109089

**Published:** 2023-05-22

**Authors:** Xue-Song Li, Jun-Ze Xue, Yu Qi, Inam Muhammad, Hao Wang, Xuan-Yu Li, Yi-Jia Luo, Dao-Mi Zhu, Yun-Hang Gao, Ling-Cong Kong, Hong-Xia Ma

**Affiliations:** 1 Department of Veterinary Medicine, College of Animal Science and Technology, Jilin Agricultural University, Xincheng Street No. 2888, Changchun 130118, Chinadr.inam@sbbu.edu.pk (I.M.);; 2 The Key Laboratory of New Veterinary Drug Research and Development of Jilin Province, Jilin Agricultural University, Xincheng Street No. 2888, Changchun 130118, China; 3 Department of Zoology, Shaheed Benazir Bhutto University Sheringal, Dir Upper 18050, Pakistan; 4 The Engineering Research Center of Bioreactor and Drug Development, Ministry of Education, Jilin Agricultural University, Xincheng Street No. 2888, Changchun 130118, China

**Keywords:** antibiotic tolerance, bacteria, citric acid, antibiotic resistance, metabolism

## Abstract

Antibiotic tolerance has become an increasingly serious crisis that has seriously threatened global public health. However, little is known about the exogenous factors that can trigger the development of antibiotic tolerance, both in vivo and in vitro. Herein, we found that the addition of citric acid, which is used in many fields, obviously weakened the bactericidal activity of antibiotics against various bacterial pathogens. This mechanistic study shows that citric acid activated the glyoxylate cycle by inhibiting ATP production in bacteria, reduced cell respiration levels, and inhibited the bacterial tricarboxylic acid cycle (TCA cycle). In addition, citric acid reduced the oxidative stress ability of bacteria, which led to an imbalance in the bacterial oxidation–antioxidant system. These effects together induced the bacteria to produce antibiotic tolerance. Surprisingly, the addition of succinic acid and xanthine could reverse the antibiotic tolerance induced by citric acid in vitro and in animal infection models. In conclusion, these findings provide new insights into the potential risks of citric acid usage and the relationship between antibiotic tolerance and bacterial metabolism.

## 1. Introduction

Our medical system has been greatly impeded by bacterial resistance to antibiotics [1]. It is worth noting that pathogenic microorganisms usually acquire antibiotic resistance through horizontal gene transfer or chromosomal mutation [2]. However, there is increasing evidence that phenotypic antibiotic tolerance has significantly reduced the efficacy of existing antibiotics [3]. Antibiotic tolerance is the ability of antibiotic-sensitive bacteria at the gene level to survive under the influence of antibiotic concentration during treatment, which plays a key role in the extended survival time of bacteria during infections [4]. Antibiotic resistance can be measured via the identification of specific genetic elements and minimum inhibitory concentration (MIC) analysis, but, in contrast, due to a dearth of clear quantitative indicators, the measurement of antibiotic tolerance lacks well-defined detection techniques. In particular, tolerance reflects the ability of microorganisms to survive under exposure to high concentrations of bactericidal substances without typical resistance mutations and increased MIC values [5]. The existence of these characteristics causes tolerant bacteria to be overlooked easily in experimental research, but their consequences remain in clinical treatment. Antibiotic tolerance plays a key role in chronic and recurrent infections [6]. Much evidence proves that despite multidrug combinations, antibiotic tolerance can promote the development and evolution of antibiotic resistance, and this situation results in treatment failure during infections [7,8]. There is still a lack of understanding of potential risk factors, such as diet and disinfection factors responsible for the development of antibiotic tolerance, both in vivo and in vitro.

With the joint approval of the ISO (International Organization for Standardization), ASTM (American Society for Testing Materials), and other national and international organizations, citric acid is widely used as a safe substance in food additives [9], animal feed [10], non-ferrous metal products [11], veterinary medicine [12], and other fields. Among these, the use of citric acid in the food and carbonated beverage industries accounts for approximately 75% of the total usage. Secondly, because citric acid has the ability to inhibit microbial growth, it is used in household detergents and cleaning agents, and even in water disinfection. According to the market report on the use of citric acid published in Japan in 2023, the global consumption of citric acid was at approximately 2.8 million tons in 2022, and it is estimated that this amount will reach up to 3.3 million tons by 2028 [13]. The ADI (acceptable daily intake) published by the FAO (Food and Agriculture Organization) and the WHO (World Health Organization) of the United Nations does not limit its use [14]. However, the widespread use of citric acid in the food industry requires a more detailed assessment of its impact on human health and the ecological environment. Previous studies have shown that the addition of organic acids such as citric acid could improve food flavor, maintain its nutritional value, and extend its shelf life [15]. At the same time, it can increase the feed intake in piglets and poultry and improve growth efficiency [16,17]. In addition, recent research has shown that citric acid can enhance the uptake of heavy metals and other substances by plants [18]. Due to the widespread use of citric acid, it naturally accumulates in the natural environment and in the human body. However, there are few reports on the possible problems in the process of citric acid addition, in particular, citric acid as a broad-spectrum antifungal affects the therapeutic response to bactericidal antibiotics, and there are few reports on the interaction between citric acid and bacterial pathogens.

In this study, we revealed that citric acid exposure to bacteria both in vitro and in vivo led to antibiotic tolerance in different bacterial species. Mechanistically, the continuous addition of citric acid in the culturing process can reduce the activity of bactericidal antibiotics by inducing the glyoxylic cycle, inhibiting the bacterial respiration rate, and weakening the intracellular oxidative stress level in bacteria. On this basis, we can effectively reverse the antibiotic tolerance caused by citric acid and restore the bactericidal activity of ciprofloxacin by supplementing with bacterial metabolites such as succinic acid and xanthine in the process of the bacterial culture and infection model of *Galleria mellonella*. Our results indicate that citric acid induced the enhancement of bacterial viability under the action of antibiotics, which was mainly due to the alteration in bacterial metabolic ability, thus endowing the bacteria with tolerance to various bactericidal antibiotics.

## 2. Results

### 2.1. Citric Acid Reduces the Killing Effect of Antibiotics on Pathogenic Bacteria

**Figure 1:**
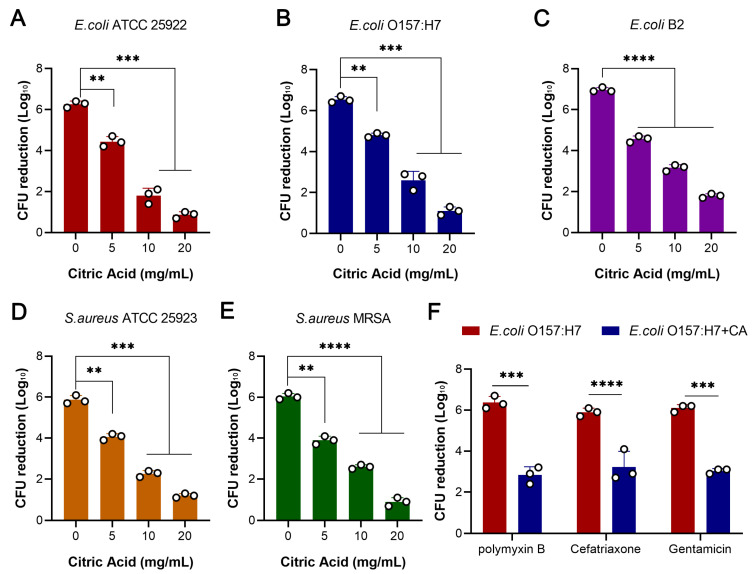
Citric acid reduces the killing activity of antibiotics against bacterial pathogens. (**A**–**E**) Citric acid co-cultured with bacteria for 6 h reduced the bactericidal activity of ciprofloxacin against Gram-positive bacteria (*S.aureus* ATCC 25923 and MRSA T144) and Gram-negative bacteria (*E. coli* ATCC 25922, *E. coli* O157:H7, and *E. coli* B2) in a dose-dependent manner. (**F**) Citric acid reduced the bactericidal activity of three bactericidal antibiotics (polymyxin B, ceftriaxone, and gentamicin). All data from biological experiments performed in triplicate are presented as means ± SD. ** *p* < 0.01, *** *p* < 0.001, and **** *p* < 0.0001, determined using non-parametric one-way ANOVA.

To investigate the effect of citric acid on bacterial growth, the MIC method was used to determine the antibacterial activity of citric acid alone against various strains. The results show that citric acid had no direct killing effect on Gram-negative and Gram-positive bacteria (MIC > 512 μg/mL) (Table S1). Then, we investigated the effect of the citric acid concentration (with a final concentration of 2%) on bacterial growth and the changes in the MICs of 5 representative strains with 7 antibiotics. The results show that adding citric acid during bacterial culture could reduce the bacterial growth and the total amount of bacteria when they reached the plateau stage (Figure S1A–E), but it did not change the MICs of seven antibiotics (Table S2). Then, we investigated the effect of ciprofloxacin on killing five susceptible pathogenic bacteria. Unexpectedly, we found that adding citric acid to the culture medium weakened the bactericidal activity of ciprofloxacin and showed dose dependency with different concentrations of citric acid (Figure 1A–E). Specifically, when the concentration of ciprofloxacin was 20 × its MIC, it could not effectively kill *E. coli* O157:H7, and the CFU decreased by approximately 1 × 10^6^. When co-cultured with 20 mg/mL of citric acid for 6 h, the CFU decreased by approximately 1 × 10^2^ and showed a 1000-fold difference in the CFU reduction. Considering that ciprofloxacin is a bactericidal antibiotic that targets DNA polymerase, we investigated whether citric acid also affected its bactericidal ability against *E. coli* O157:H7 under the different bactericidal mechanisms of different antibiotics such as polymyxin B, ceftriaxone, and gentamicin. The results show that citric acid could reduce the activity of three bactericidal antibiotics (Figure 1F). 

The addition of citric acid can change pH, as pH is an important factor that affects bacterial growth and the bactericidal activity of antibiotics. In order to explore the main reasons for the antibiotic tolerance caused by citric acid, acetic acid (an organic acid) and hydrochloric acid (an inorganic acid) were also set up, and these 3 acids were added to keep the pH of the medium consistent (pH = 2.2). The bactericidal activity of ciprofloxacin, polymyxin B, ceftriaxone, and gentamicin against *E. coli* O157:H7 was observed while adding the different acids. We found that the addition of acetic acid could reveal the same results as citric acid, but the addition of hydrochloric acid had little effect on the bactericidal activity of the four bactericidal antibiotics (Figure S1F). This may be because hydrochloric acid is an inorganic acid. Although the addition of hydrochloric acid changed the pH of the whole environment, unlike citric acid, it has no bactericidal ability against bacteria. These results indicate that citric acid weakens the activity of bactericidal antibiotics against pathogenic bacteria.

### 2.2. Citric Acid Can Induce Heritable Antibiotic Tolerance in Bacteria

**Figure 2:**
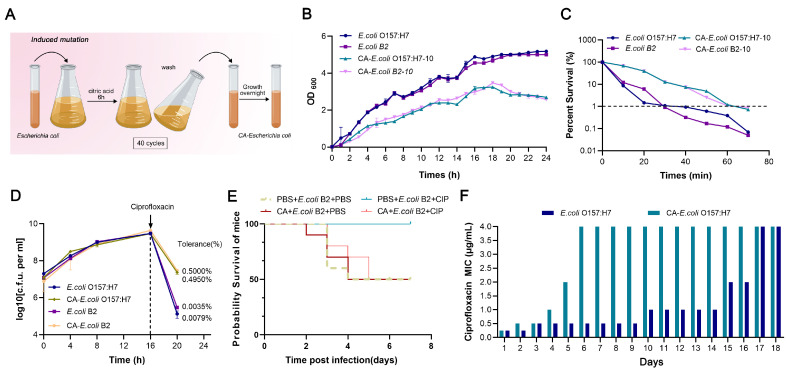
Citric acid can induce heritable antibiotic tolerance in bacteria in vivo and in vitro. (**A**) Wild-type *Escherichia coli* (*E. coli*) was grown continuously under citric acid pressure for 40 generations to obtain tolerant *Escherichia coli* (CA-*E. coli*) (**B**) The optical density (OD_600_) values of *E. coli* and CA-*E. coli* were monitored over 24 h. (**C**) The MDK_99_ test was used to determine the survival rates of *E. coli* and CA-*E. coli* strains over 60 min to assess their antibiotic tolerance. (**D**) A high concentration of ciprofloxacin (20 × MIC) was added to the *E. coli* and CA-*E. coli* bacterial solutions after 16 h of incubation, and the frequency of antibiotic tolerance formation in the bacteria was determined. (**E**) Survival rates of mice infected with *E. coli* B2 and CA-*E. coli* B2 treated with PBS or ciprofloxacin (50 mg/kg). Female BALB/c mice (n = 10 per group) were given PBS and citric acid for 5 days before infection, followed by *E. coli* B2 and CA-*E. coli* B2 (1.0 × 10^8^ CFUs), and a single dose of ciprofloxacin (50 mg/mL) 1 hour after infection. (**F**) The times taken for the *E. coli* O157:H7 and CA-*E. coli* O157:H7 strains to develop drug resistance were measured by continuously increasing the concentration of ciprofloxacin in vitro. This experiment was replicated three times.

From the acquisition of tolerant strains in Figure 2A, we know that antibiotic tolerance is usually accompanied by a reduction in bacterial growth activity. Measuring the growth curves of the induced strains, the *E. coli* O157:H7 strain (CA-*E. coli* O157:H7) and the *E. coli* B2 strain (CA-*E. coli* B2), induced with citric acid, showed significantly slower growth rates in the 10th generation, and the total numbers of bacteria decreased when they reached plateaus (Figure 2B). The MDK_99_ test (the minimum duration for killing 99% of cells) showed that the time required for the 10th-generation strain to reduce its number to 1 × 10^2^ was prolonged by 30 min (Figure 2C), but the MIC did not change (Table S3). This indicates that the 10th-generation strain had apparent antibiotic tolerance under citric acid induction. After 40 generations of induction, the citric acid was removed from the culture conditions. After 20 generations of continuous culturing on Mueller–Hinton agar (MHA) without antibiotics, antibiotic tolerance was still present (Figure S2A,B). At the same time, by measuring the frequency of tolerance formation in *E. coli* O157:H7 and *E. coli* B2 before and after tolerance induction, it was found that the frequency of tolerance formation in the CA-*E. coli* B2 strain increased by approximately 62-fold and that in the CA-*E. coli* O157:H7 strain increased by approximately 142-fold after treatment with ciprofloxacin for 4 h (Figure 2D). These results indicate that citric acid can induce heritable antibiotic tolerance in bacteria.

It was demonstrated that citric acid can induce antibiotic tolerance in bacteria in vitro; therefore, we proceeded to observe whether citric acid could confer bacterial tolerance in vivo and reduce the clinical therapeutic effects of antibiotics. We first observed the in vivo efficacy of ciprofloxacin in an acute infection model in mice. Observing the survival rates in the control group and the citric acid supplementation group, ciprofloxacin was less effective in the citric acid supplementation group (Figure 2E). This suggests that citric acid may play a role in the mice infection model that did not reach therapeutic concentrations but had the same effect as bactericidal antibiotics.

### 2.3. Tolerant Strains Induced with Citric Acid Were More Likely to Develop Drug Resistance

By using ciprofloxacin to induce resistance in *E. coli* O157:H7 and CA-*E. coli* O157:H7 and to monitor the time for the two strains to reach the drug resistance breakpoint, we found that the CA-*E. coli* O157:H7 strain developed drug resistance faster. Specifically, the MIC of the CA-*E. coli* O157:H7 strain increased rapidly in the 3rd generation and reached the CLSI resistance breakpoint (MIC > 4 μg/mL) in the 6th generation. The MIC value of the *E. coli* O157:H7 strain increased slowly and reached the drug resistance breakpoint in the 17th generation, which was 11 generations later than that of the tolerant strains (Figure 2F).

### 2.4. Transcriptome and Intracellular Metabolite Analyses of E. coli Induced with Citric Acid

**Figure 3:**
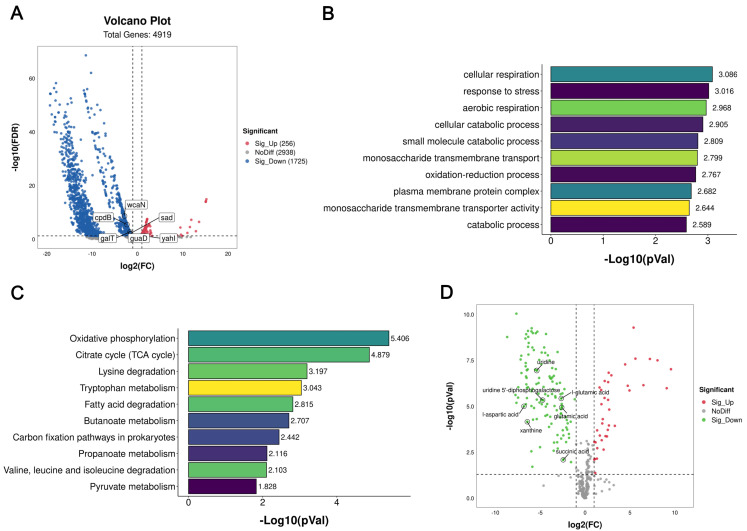
Transcriptional and metabolic responses of *E. coli* O157:H7 strain before and after antibiotic tolerance induced with citric acid. (**A**) Transcriptome volcanic maps. (**B**) Biological process of transcriptome GO. (**C**) Transcriptome KEGG pathway. (**D**) Metabolomic volcanic map. The x-axis and y-axis in (**A**,**D**) indicate the degree of expression change and statistical significance, respectively. In (**B**,**C**), the y-axis is the name of the significantly enriched pathway, and the x-axis indicates the level of significance. Log10 fold changes are shown.

To explore the molecular mechanism of citric-acid-induced antibiotic tolerance, we analyzed the transcriptomes of the tolerant and susceptible *E. coli* O157:H7 strains. The results show that there were a number of differentially expressed genes (DEGs) in the tolerant *E. coli*, including 256 up-regulated genes and 1725 down-regulated genes (with a fold change of ≥ 2-fold; Figure 3A). Significant changes occurred in the GO enrichment analysis, including significant changes in gene pathways such as cell respiration and the response to stress (Figure 3B). A KEGG pathway analysis showed that the citrate cycle (TCA cycle) and oxidative phosphorylation were related (Figure 3C). mRNA expression of DEGs was detected using RT-qPCR (Figure S2C) and was consistent with the transcriptome results.

A total of 603 differentially expressed products were found in non-target metabolites, of which 188 products were up-regulated and 515 products were down-regulated (Figure 3D). Through a correlation analysis between transcriptome sequencing and metabonomic sequencing, we focused on the analysis of differential genes and metabolites enriched in the same metabolic pathway. By analyzing the correlation results, a total of six differential genes and their metabolites that could affect tolerance, and which were related to galactose metabolism, arginine synthesis, aspartate and glutamate metabolism, and pyrimidine metabolism, were screened for subsequent functional verification. Based on these results, we speculate that citric-acid-induced antibiotic tolerance might be related to the TCA cycle, cellular respiration, and oxidative stress.

### 2.5. Citric Acid Causes Bacterial Tolerance by Reducing Bacterial Energy Metabolism

**Figure 4:**
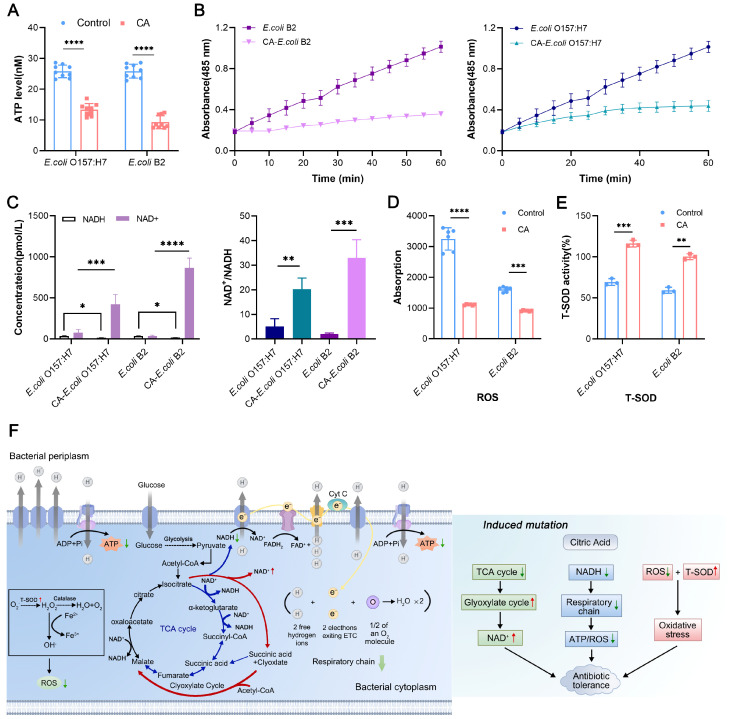
Mechanism of citric-acid-induced antibiotic tolerance in *E. coli*. (**A**): The *E. coli* and CA-*E. coli* strains produced intracellular ATP. Intracellular ATP production in the tolerant strain was significantly reduced. (**B**): Intracellular respiration was measured in the *E. coli* and CA-*E. coli* strains. The levels of intracellular respiration in the antibiotic-tolerant strains were significantly reduced. (**C**): After citric acid induced antibiotic tolerance, it led to the accumulation of NAD^+^, a decrease in NADH, and an increase in the NAD^+^/NADH ratio. (**D**,**E**): After inducing antibiotic tolerance, citric acid led to a decrease in intracellular ROS production and accumulation of T-SOD, which induced the bacterial oxidative stress response. (**F**): Schematic diagram of the mechanism by which citric acid protects bacteria from being killed by antibiotics. In the figure, the red up arrow indicates an increase in yield or activation of related pathways, while the green down arrow indicates a decrease in yield or inhibition of related pathways. All data from biological experiments performed in triplicate are presented as means ± SD. * *p* < 0.05, ** *p* < 0.01, *** *p* < 0.001, and **** *p* < 0.0001, determined using non-parametric one-way ANOVA.

Using specific fluorescent probes, we found that citric-acid-induced tolerance of *E. coli* did not affect its outer membrane integrity and permeability and did not alter the ability to form a biofilm. However, when we observed the cell membrane potential, we found that the membrane potential of tolerant bacteria was significantly dissipated (Figure S3A–D). Membrane potential is an important component of bacterial proton dynamics and an important requirement for ATP synthesis from ADP + Pi. The dissipation of the membrane potential suggests that ATP production might have been affected. At the same time, based on the transcriptome and metabonomics sequencing results, we speculate that citric acid may alter the normal energy metabolism of bacteria after tolerance induction. To confirm this, we measured the intracellular ATP production of bacteria, and the intracellular ATP content decreased in the citric-acid-induced tolerant strains (Figure 4A). It is well known that ATP depletion can lead to the production of tolerant cells of *E. coli* and *S. aureus*, and ATP is the energy source for aerobic respiration in cells as well [19].

Therefore, we measured the respiration levels in the sensitive and tolerant strains, and the results show that within 60 min, the cell respiration level in the tolerant bacteria was lower than that in the sensitive strains (Figure 4B). All these results indicate that the energy metabolism activity in the bacteria was inhibited. It is well known that the main site of energy metabolism in bacteria is in the TCA cycle, which involves a dehydrogenation reaction to produce three NADH molecules as electron donors for cell respiration. By detecting NAD^+^ and the NAD^+^/NADH ratio, the accumulation of NAD^+^ in the tolerant strains was shown to be accompanied by an increase in the NAD^+^/NADH ratio, while the production of NADH decreased (Figure 4C), indicating that the TCA cycle in the tolerant bacteria was inhibited, which may have directly affected the activation of the glyoxylate cycle.

The endogenous ROS in bacteria is produced via the electron transport chain of aerobic respiration. We determined the endogenous ROS in the sensitive bacteria and tolerant bacteria and found that the endogenous ROS production in the tolerant bacteria was significantly decreased (Figure 4D). Based on the previous transcriptome analysis, it has been shown that antioxidant-related genes are differentially expressed in tolerant strains. Therefore, we evaluated the changes in T-SOD expression in the sensitive and tolerant strains. SOD is an effective scavenger of free radicals in bacteria and can produce H_2_O_2_ in bacteria and thus provide cell defense against oxidative damage. By measuring the intracellular T-SOD levels, we found that the T-SOD levels in the tolerant strain were significantly higher than those in the sensitive strains (Figure 4E). These results indicate that citric acid reduced the activity of bactericidal antibiotics in a variety of ways, including reducing bacterial energy metabolism, inhibiting bacterial respiration, inhibiting the TCA cycle, activating the glyoxylate cycle, and enhancing the bacterial antioxidant response (Figure 4F).

### 2.6. Exogenous Metabolites Can Reverse the Bactericidal Activity of Antibiotics against Tolerant Bacteria

The correlation analysis of the transcriptome and non-target metabolomics was screened for six genes (*sad*, *galT*, *guaD*, *cpbB*, *yahI*, and *gabD*) that can lead to antibiotic tolerance. By analyzing the differentially expressed genes in the *E. coli* O157:H7 strain and the CA-*E. coli* O157:H7 strain, the gene deletion strain, the gene-deletion-complemented strain, and the gene overexpression strain were constructed to identify the gene functions, respectively. Changes in the bactericidal activity of ciprofloxacin were then observed in all the gene-edited strains. We found that both knockout of the *guaD* gene and overexpression of the *gabD* gene in the *E. coli* O157:H7 strain could induce antibiotic tolerance. Similarly, overexpression of *guaD* or *gabD* knockout in CA-*E. coli* O157:H7 could restore some susceptibility in CA-*E. coli* O157:H7 (Figure S4). The *guaD* gene was a key gene in the association analysis for the regulation of xanthine oxidase in purine metabolism. The *gabD* gene mainly regulated the production of succinic acid. In short, in the tricarboxylic acid (TCA) cycle, succinic acid production was significantly down-regulated, while the *gabD* gene, a key regulatory gene of the succinic-acid-degrading enzyme, was significantly upregulated. At the same time, it is worth mentioning that the *aceB* gene encoding malate synthase was extremely up-regulated, which means that the glyoxylate cycle may have been activated to complement the TCA cycle and show an adaptive metabolic response to the external environment. In the purine pathway, the production of xanthine, a key product, decreased significantly, while the *guaD* gene, a key gene for xanthine synthesis, also showed significant down-regulation. The *guaD* gene encodes guanine deaminase, which catalyzes the hydrolysis and deamination of guanine and xanthine synthesis. The process of xanthine metabolism in bacteria produces excessive hydrogen peroxide, which leads to a change in bacterial oxidative stress. 

**Figure 5:**
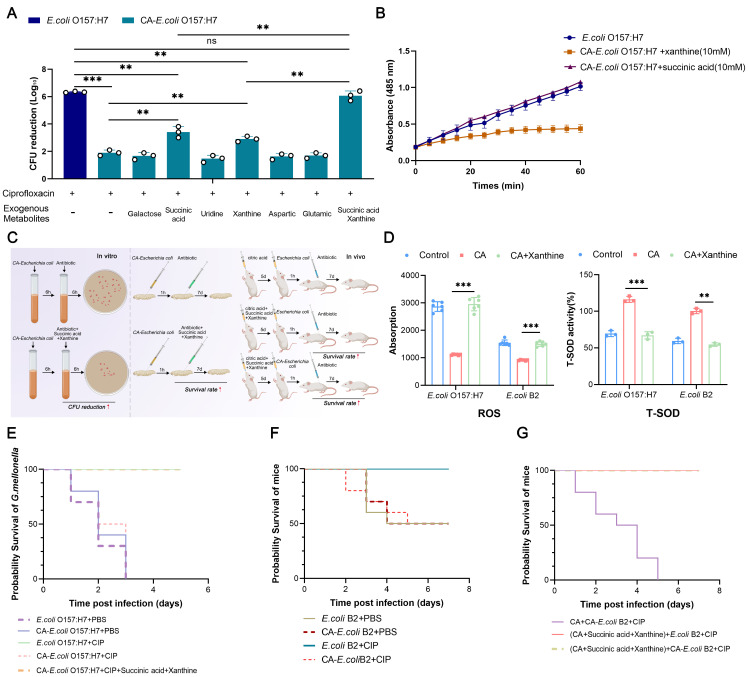
The addition of exogenous metabolites can reverse citric-acid-induced antibiotic tolerance and restore the activity of bactericidal antibiotics. (**A**) Bactericidal activity of ciprofloxacin against *E. coli* O157:H7 and CA-*E. coli* O157:H7 strains in the presence or absence of six metabolites. (**B**) Altered levels of intracellular respiratory capacity of *E. coli* O157:H7 and CA-*E. coli* O157:H7 strains in the presence or absence of succinic acid and xanthine. (**C**) The combination of succinic acid and xanthine with ciprofloxacin can enhance the bactericidal activity of ciprofloxacin against resistant bacteria in vitro and in vivo. (**D**) Intracellular ROS and T-SOD production capacity of *E. coli* O157:H7 and CA-*E. coli* O157:H7 strains in the presence or absence of succinic acid and xanthine. (**E**) Survival statistics of *E. coli* O157:H7 infected G. mellonella (n = 10 per group) with or without ciprofloxacin and ciprofloxacin in combination with metabolites. (**F**) Survival of mice (n = 10 per group) infected with PBS or ciprofloxacin (50 mg/mL) and treated with *E. coli* B2 and CA-*E. coli* B2 strains (3.0 × 10^8^ CFUs). (**G**) Survival rate of mice infected with *E. coli* B2 and CA-*E. coli* B2 strains treated with PBS or ciprofloxacin (50 mg/kg). Female BALB/c mice (n = 10 per group) were given citric acid or combination of citric acid and metabolites for 5 days before infection, followed by *E. coli* B2 and CA-*E. coli* B2 (1.0 × 10^8^ CFUs), and a single dose of ciprofloxacin (50 mg/mL) 1 hour after infection. All data from biological experiments performed in triplicate are presented as means ± SD. ** *p* < 0.01, *** *p* < 0.001, and determined using non-parametric one-way ANOVA. NS indicates not significant.

There is considerable evidence that remodeling bacterial metabolism is a new strategy for eradicating bacteria that are difficult to treat with antibiotics. We, therefore, investigated whether citric-acid-induced bacterial tolerance could be reversed via the addition of exogenous metabolites. To validate this view, we used the sensitive and tolerant strains in combination with six metabolites (galactose, succinic acid, uridine, xanthine, aspartic, and glutamic) during ciprofloxacin treatment. Interestingly, we found that when succinic acid and xanthine were used alone, the killing effect of ciprofloxacin on the tolerant bacteria was enhanced but could not be restored to its level in the sensitive strains. However, when succinic acid and xanthine were combined with the tolerant bacteria, the killing effect of ciprofloxacin on the tolerant bacteria was reversed and restored to its level in the susceptible strains (Figure 5A). However, the addition of the other four metabolites did not improve the bactericidal activity of the bactericidal antibiotics. We then investigated the molecular mechanism of succinic acid and xanthine in restoring bacterial tolerance. By measuring the respiration levels in bacteria and the levels of ROS and T-SOD in cells 60 min after the metabolites were added, we found that succinic acid could restore the respiration level in the tolerant bacteria (Figure 5B), and xanthine oxidase could restore the oxidative stress level in the tolerant strains (Figure 5D). These phenomena tell us that citric-acid-induced antibiotic tolerance can be reversed when specific exogenous metabolites are added. 

### 2.7. Exogenous Metabolites Can Improve the Therapeutic Effect of Bactericidal Antibiotics In Vivo

We found that succinic acid and xanthine reversed citric-acid-induced tolerance in vitro, and we then tested the clinical efficacy of these two metabolites in an infection model of *Galleria mellonella* and mice (Figure 5C). We found that the survival rates in *Galleria mellonella* and mice infected with the sensitive strains or antibiotic-tolerant strains were significantly improved whether they were used in combination with metabolites during ciprofloxacin treatment or with added metabolites alone during citric acid treatment (Figure 5E–G). This suggests that succinic acid and xanthine can be used as adjuvants to prevent the formation of antibiotic-tolerant strains during the use of citric acid and can also be used in combination with antibiotics to kill antibiotic-tolerant strains. 

These data demonstrate that citric acid can induce antibiotic tolerance in bacteria and that the addition of some specific exogenous metabolites can effectively reverse this process, both in vivo and in vitro.

## 3. Discussion

The discovery and application of antibiotics is one of the great discoveries of mankind since the 20th century [20]. However, bacteria have gradually evolved various drug resistance and tolerance mutations to counteract the killing effect of antibiotics [21]. The common mechanism of drug resistance is the presence of drug resistance genes in bacteria and their intraspecific transmission mediated via plasmids [2,22]. In recent years, the antibiotic tolerance of bacteria has attracted worldwide attention, and its emergence has greatly reduced the clinical therapeutic effect of antibiotics [3,23]. Worryingly, the reason antibiotic tolerance is easily overlooked is the lack of effective characterization indicators [5,24]. To date, some molecules related to bacterial metabolism have been shown to induce antibiotic tolerance and reduce antibiotic efficacy. NO [25], H2S [26], indole [27], phenazine [28], and some major components of disinfectants can reduce the bactericidal activity of antibiotics by alleviating oxidative damage and reducing cell respiration or metabolic activity [29]. However, during this period, it was also discovered that by altering the metabolic capacity of bacteria, they can restore the ability of bactericidal antibiotics [30]. Nevertheless, there are still many undiscovered risks in the relationship between human physiological activities and the development of antibiotic tolerance.

In this study, we found that citric acid, which is widely used in many fields, can reduce the killing effect of bactericidal antibiotics after exposure to bacteria, and it shows a certain dose dependency with an increasing concentration. Citric acid, an organic acid with bactericidal ability, has an antibacterial mechanism that mainly involves disrupting the cell membrane of bacteria and lowering the pH to arrest bacterial growth. Herein, we have discussed whether pH reduction is a key factor in the development of antibiotic tolerance. By setting up acetic acid (an organic acid) and hydrochloric acid (an inorganic acid) alongside citric acid, we found that acetic acid with bactericidal ability had the same potential to reduce the bactericidal activity of antibiotics as citric acid did, but hydrochloric acid with bacteriostatic ability did not lead to changes in the bactericidal activity of antibiotics. These results suggest that pH alteration is not a key factor in the development of antibiotic tolerance; rather, the key factor to induce antibiotic resistance in bacteria is the bactericidal ability of citric acid against bacteria similar to that of bactericidal antibiotics.

At the same time, we simulated the continuous exposure of bacteria to citric acid in everyday use and found that the long-term presence of citric acid can induce bacteria to develop heritable antibiotic tolerance. Compared with normal cells, these induced bacteria have no obvious changes in MIC, confirming that citric acid leads to the development of antibiotic tolerance rather than antibiotic resistance. It is worth noting that the clinical efficacy of ciprofloxacin in the acute infection model was reduced by feeding the mice a relatively high dose of citric acid in advance, indicating that citric acid can indeed induce antibiotic tolerance both in vivo and in vitro.

The mode of action of citric acid is to reduce the energy metabolism of bacteria, which ultimately leads to the production of tolerance. The main expression of this is a reduction in total ATP production and the inhibition of succinic acid synthesis in the TCA cycle, thus activating the glyoxylate cycle. The highly significant up-regulation of the *aceB* gene found in the transcriptome results also confirmed the activation of the glyoxylate cycle. This pathway is accompanied by a decrease in NADH (the respiratory chain supplier), which ultimately leads to a decrease in bacterial respiration levels [31]. These findings also support the hypothesis that activation of the glyoxylate cycle may lead to the production of tolerant bacteria. Similarly, the use of isocitric acid or other citrates in daily additives during the production of citric acid, an important product in the TCA cycle, may likewise have an impact on antibiotic tolerance, which is an area of concern in our future studies. At the same time, ROS production in tolerant bacteria decreased, and ROS-mediated oxidative damage might be an auxiliary way for antibiotics to kill bacteria [32]. Likewise, at the same time, the production of T-SOD, a key substance that alleviates bacterial oxidative damage, increased significantly, which might have led to excessive production of free radicals in bacterial cells, and this series of typical oxidative stress characteristics were activated, including DNA damage and protein synthesis disorder [33].

In studying antibiotic tolerance, we often need to distinguish it from bacterial persistence. Persistence is the ability of bacteria to survive as a small subpopulation of bacteria under high concentrations of antibiotic treatment, and after antibiotic pressure subsides, the persistent bacteria resume growth and regain their antibiotic-sensitive state [34,35]. In contrast, tolerant bacteria survive longer than sensitive bacteria under antibiotic pressure, but they are not unable to be killed, and their population density decreases slowly. Moreover, in terms of numbers, persistent bacteria usually make up approximately 1/10,000 of the bacterial population, while tolerant bacteria make up a much larger percentage of the population, reaching up to approximately 1/100 [36]. According to previous reports [3,8,30,37,38], neither persistent nor tolerant bacteria are usually accompanied by the mutational selection of bacterial genes, and persistent and tolerant bacteria are also usually acquired through the preservation of tolerant bacterial subpopulations throughout the bacterial community in the presence of persistent antibiotic pressure, along with the ability to be genetically stable. In our study, we hypothesized that under the pressure of the continuous presence of citric acid, the tolerant bacterial subpopulation would be retained and become the main body of the bacterial population, which is why we were able to use citric acid in vitro, which could successfully induce the production of tolerant bacteria.

A large number of studies have shown that the production of tolerant bacteria can be reversed by altering the metabolic activity of bacteria [28,39,40,41,42]. Therefore, activating their metabolic activity is a new and reasonable way to combat antibiotic tolerance. For example, studies have shown that cysteine and thymine improve the activity of bactericidal antibiotics by increasing bacterial respiration levels and oxidative damage [30]. At the same time, tolerance to antibiotics can be increased by increasing the levels of ATP-dependent dynamic protein [19]. Considering that citric-acid-induced tolerance is closely related to bacterial metabolism, we believe that improving bacterial metabolic activity is the strategy to restore citric-acid-induced tolerance. Using this knowledge, we found that succinic acid and xanthine can effectively reverse tolerance and restore the therapeutic effects of antibiotics in vitro and in animal infection models.

In short, citric acid activated the glyoxylate cycle, reduced the level of cellular respiration, induced an imbalance in the intracellular oxidation system, and activated antibiotic tolerance in bacteria both in vitro and in vivo. Importantly, by adding exogenous metabolites, we found that succinic acid and xanthine rescued the efficacy of bactericidal antibiotics. These findings suggest that the use of citric acid in food, chemicals, feed, and other items requires increased attention. At the same time, it may play a more important role in the fight against tolerant pathogens through the addition of combined adjuvants that activate bacterial metabolism.

## 4. Materials and Methods

### 4.1. Bacterial Strains and Chemical Reagents

All strains used in this study are listed in Table S4. Unless otherwise indicated, all strains were routinely resuspended on Mueller–Hinton agar (MHA) and cultured aerobically in Mueller–Hinton broth (MHB) at 37 °C and 160 rpm. Solid antibiotics were purchased from the China Veterinary Drug Research Institute, diluted to 20,480 μg/mL according to CLSI standards, and stored at −20 °C for later use, and other reagents were purchased from Sigma-Aldrich (Sigma Aldrich Trading Co., Ltd., Shanghai, China) or Beyotime Biotechnology (Beyotime Biotech. Inc., Shanghai, China).

### 4.2. Minimum Inhibitory Concentration (MIC) Determination

In accordance with CLSI 2022 guidelines, the bactericidal activity of seven bactericidal antibiotics and citric acid against five bacterial strains was evaluated using the MIC test. Briefly, in a sterile 96-well plate, the drugs were continuously serially 2-fold-diluted in MHB and then mixed with an equal volume of bacterial suspension with a bacterial concentration of 1 × 10^6^ CFU/mL. After incubation at 37 °C for 16 h, the MICs of the drugs were determined using a 96-well microplate reader. Each test was performed in triplicate.

### 4.3. Effect of Citric acid on Bactericidal Activity of Antibiotics and Bacterial Growth

The *S. aureus* ATCC 25923, MRSA T144, *E. coli* ATCC 25922, *E. coli* O157:H7, and *E. coli* B2 strains that were grown overnight were diluted at 1:1000 in 5 mL of fresh MBH medium containing a 2% final citric acid concentration or no citric acid. The cultures were incubated in a shaking flask at 37 °C and 160 rpm. Samples were taken at every 2 h interval, sterile MHB was used as a blank control, and the values of different culture times were measured at the wavelength of OD_600_ nm. The curves were plotted with culture time as the abscissa and OD_600_ nm as the ordinate, and three replicates were set up in the experiment.

Then, the *S. aureus* ATCC 25923, MRSA T144, *E. coli* ATCC 25922, *E. coli* O157:H7, and *E. coli* B2 strains that were cultured overnight were diluted at 1:100 in 1 mL of fresh MBH medium containing different concentrations of citric acid (0, 5, 10, and 20 mg/mL). They were then cultured on a shaking table at 37 °C and 160 rpm. After 6 h, the bacteria were collected via centrifugation, washed with PBS 3 times, and then suspended in 1mL of MHB. A 50 μL bacterial solution was taken out and plated on an MHA plate, and the colonies were counted. Then, a ciprofloxacin solution was added to make the final concentration of the drug in the culture medium 20 × MIC of the corresponding bacteria. After 6 h of adding the solution, 50 μL of the bacterial solution was taken out and diluted with a serially 10-fold ratio, the colony count was carried out, and the corresponding colony value difference was calculated to express the bactericidal activity of the drug. Then, polymyxin B, ceftriaxone, and gentamicin were selected to determine the bactericidal activity according to the above steps, and the procedure was repeated three times.

Then, the *E. coli* ATCC 25922, *E. coli* O157:H7, and *E. coli* B2 strains that were grown overnight were diluted at 1:100 in 1mL of fresh MBH medium containing 20 mg/mL of citric acid or no citric acid. The final concentration of ciprofloxacin was 20 × MIC of the corresponding strain, and the colonies were counted after 50 μL was taken every 10 min. The time to kill 99% of the bacteria (MDK_99_) was observed. The curve was plotted with time as the abscissa and colony count as the ordinate. The experiment was repeated three times.

### 4.4. Citric Acid Induces Antibiotic Tolerance in Bacteria

The *E. coli* O157:H7 and *E. coli B2* strains that were grown overnight were inoculated at 1:10 into 50 mL of MHB containing 20 mg/mL and grown on a shaking table at 37 °C and 200 rpm. After 6 h, citric acid was removed via centrifugation, they were washed with PBS three times, and then they were suspended in 5 mL of MHB for 16–18 h. This was used as a cycle for 40 times of continuous culture and induction, and 3 parallel groups were established. According to the above method, after every five cycles, the strains that were cultured overnight without citric acid were collected, and the changes in the MIC, growth rate, and MDK_99_ of the strains were measured to determine the formation of antibiotic tolerance induced by citric acid.

The *E. coli* O157:H7 and *E. coli B2* strains that were cultured overnight before and after induction were diluted at 1: 100 in MHB at 37 °C and 160 rpm and cultured for 16 h, and the colonies were counted. Then, a ciprofloxacin solution was added, whereby the final concentration of the solution was 20 × MIC, 37 °C, and they were centrifuged at 160 rpm and cultured for 4 h, and the remaining colonies after ciprofloxacin action were counted, which was termed the frequency of tolerance formation in bacteria.

### 4.5. Drug Resistance Formation Time

According to the CLSI 2022 test requirements, the time for resistance formation in *E. coli* O157:H7 strain before and after induction was measured by increasing the concentration of ciprofloxacin in vitro. Briefly, 5 mL of MHB containing ½ the MIC of ciprofloxacin with 1% inoculum was shaken at 37 °C for 12 h each time. In case of bacterial growth, a 50 μL aliquot was taken out and inoculated into an MHB containing the MIC and incubated at 37 °C for 12 h. If there was no growth, the previous generation was selected for further cultivation, and the record algebra was increased by 1 generation until the level of drug resistance was above the CLSI clinical drug resistance tipping point (4 μg/mL).

### 4.6. Transcriptomic Analysis

Before and after induction, *E. coli* O157:H7 was cultured in MHB containing 20 mg/mL of citric acid or without citric acid for 6 h to the logarithmic phase. Then, the bacteria were washed with sterile PBS 3 times. Total RNA was extracted using the TRIzol Reagent kit (Invitrogen, Thermo Fisher Scientific Inc., Shanghai, China), and rRNA was removed using the Ribo-Zero Magnetic kit (EpiCentre, Beijing Baiao Innovation Technology Co., Ltd., Beijing, China). Subsequently, RNAs were converted into a cDNA library with the UNG enzyme and sequenced using the Illumina TruseqTM RNA sample prep kit system. The original data were controlled with Qualimap software(2.2.1) and compared with the *E. coli* O157:H7 genome. RPKM (reads per kilobase of transcript per million reads mapped) was used for differentially expressed genes. A gene expression level was analyzed when the *p*-value was ≤0.5 and the FC value was ≥2.0, and edgeR software(R3.6.3) was used for differentially expressed genes. Gene Ontology (GO) and Kyoto Encyclopedia of Genes and Genomes (KEGG) enrichment analyses were performed using Goatols (https://github.com/tanghaibao/GOatols, accessed on 7 November 2022).

### 4.7. Quantitative Reverse Transcription-PCR (RT-qPCR) Analysis

The *E. coli* O157:H7 strain was cultured overnight in MHB before and after induction. Total RNA was extracted using TRIzol™ Max™ (Thermo Fisher Scientific), and the absorbance (260 nm/280 nm) was measured with a spectrophotometer (Thermo Fisher Scientific). Before cDNA synthesis, the extracted RNA was adjusted to the same concentration with sterile water. Reverse transcription was performed on 1 μg of the total RNA using the PrimeScript™ RT kit (Takara, TaKaRa Biotechnology (Dalian) Co. Ltd., Dalian, China).

The mRNA levels of all representative genes were expressed as 2^−ΔΔCt^ relative to the control genes (16S RNA) in *E. coli*. A list of the optimized primers used in the RT-qPCR analysis is provided in Table S5. A standard 2-step PCR amplification standard procedure was performed with the following conditions: 95 °C for 30 s, 40 cycles at 95 °C for 5 s, and 60 °C for 34 s. The RT-qPCR test was performed using the 7500 Fast Real-Time PCR System (Applied Biosystem, Thermo Fisher Scientific).

### 4.8. LC-MS/MS Analysis

Bacterial metabolites were quenched by immersing the bacterial culture in a precooled mixture of acetonitrile, methanol, and water with a 40:40:20 ratio, respectively. The cells were physically disrupted via centrifugation at 12,000 rpm for 10 min at 4 °C. The supernatant was evaporated to less than 1 mL under nitrogen at 4 °C and redissolved with 1 mL of acetonitrile. After centrifugation at 12,000 rpm for 10 min and filtration through a 0.2 µm filter, the clarified samples were prepared for subsequent LC-MS/MS analysis.

We used a high-resolution Q Exactive mass spectrometer (Thermo Fisher Scientific USA) and LC-MS/MS technology to collect positive ion (pos) and negative ion (neg) for the detection, identification, and quantification of metabolites through non-target metabonomics. Compound Discoverer 3.1.0 (Thermo Fisher Scientific, USA) software was used for data processing. Metabolite annotation, classification (KEGG, HMDB), and enrichment analysis of identified substances were carried out by using an in-house metabonomics database to explain the physical and chemical properties and biological functions of the metabolites. The differential metabolites were screened based on the VIP values of the first two principal components of the partial least squares discriminant analysis (PLS-DA) and the results of the Fold change and Student’s *t*-test of the univariate analysis.

### 4.9. Functional Verification of Differentially Expressed Genes

Referring to Sung’s method [43], the *sad*, *galT*, *guaD,* and *cpbB* genes of the *E. coli* O157:H7 strain were knocked out using the λ-Red gene deletion strategy. The *yahI* and *gabD* genes of the CA-*E. coli* O157:H7 strain were deleted. According to the report by Shi et al. [44], the pBBR plasmid was used to construct *sad*, *galT*, *guaD*, *cpbB* gene-deletion-complemented strains and *yahI* and *gabD* gene overexpression strains of the *E. coli* O157:H7 strain. Similarly, the CA-*E. coli* O157:H7 strain overexpressing *sad*, *galT*, *guaD*, *cpbB*, *yahI*, and *gabD*, and *yahI* and *gabD* gene-deletion-complemented strains were constructed simultaneously.

### 4.10. Biochemical Parameters Assay

#### 4.10.1. Outer Membrane Permeability

The fluorescent probe 1-N-phenylnaphthylamine (NPN) (10 μM) was used and served as an indicator to evaluate the outer membrane permeability of *E. coli* O157:H7 and *E. coli* B2. Fluorescence units were measured with an excitation wavelength of 350 nm and an emission wavelength of 420 nm.

#### 4.10.2. Cell Membrane Integrity

The fluorescent probe propidium iodide (PI) (0.5 μM) was used to assess the cell membrane integrity of *E. coli* O157:H7 and *E. coli* B2, and the fluorescence units were immediately measured at an excitation wavelength of 535 nm and an emission wavelength of 615 nm.

#### 4.10.3. Membrane Depolarization

The fluorescent probe 3,3′-dipropylthiadicarbocyanine iodide DiSC3(5) was added to the bacterial cultures at 0.5 μM for detection, and real-time changes in membrane potential were monitored using an Infinite M200 microplate reader with an excitation wavelength of 622 nm and an emission wavelength of 670 nm

#### 4.10.4. Biofilm Formation

An amount of 200 μL of the strains with an OD_600_ of 0.4–0.6 was added to a 96-well plate, each sample was repeated 6 times, and MHB was added as a negative control. After incubation at 37 °C for 24 h, the culture medium was discarded, and the samples were washed 4 times with PBS. The samples were then fixed with formaldehyde for 25 min, and the medium was discarded again. Crystal violet dye was added for 25 min, and the absorbance was measured at 595 nm.

#### 4.10.5. Efflux Pump Suppression Test

According to CLSI 2022, carbonyl cyanide m-chlorophenyl hydrozone (CCCP) and verapamil (VP), two commonly used efflux pump inhibitors, were diluted to a concentration of 100 μM and stored in a refrigerator at −20 °C for later use. The method of Liu Y et al. [30] was used to determine whether the addition of an efflux pump inhibitor affects the bactericidal activity of ciprofloxacin against *E. coli* and CA-*E. coli*.

#### 4.10.6. Determination of Intracellular ATP 

The intracellular ATP levels in *E. coli* O157:H7 and *E. coli* B2 were determined by using an Enhanced ATP Assay Kit (Beyotime). The bacterial cultures were centrifuged at 12,000 rpm for 5 min at 4 °C and the supernatants were removed. Meanwhile, the bacterial precipitates were lysed with ATP lysate to release the intracellular ATP within a sufficient lysis time of up to 10 min, and then the solutions were centrifuged, and the supernatants were prepared for the measurement of ATP levels. An amount of 100 μL of the detection solution and 20 μL of the supernatants were added to the wells and rapidly mixed, and the total ATP levels were calculated from the luminescence signals accordingly. 

#### 4.10.7. Determination of NAD^+^/NADH 

Before and after induction, *E. coli* O157:H7 and *E. coli* B2 cells with an OD_600_ of 0.5 were mixed with a ciprofloxacin solution at a final concentration of 20 × MIC in MHB. After 6 h of incubation at 37 °C and 160 rpm, the cells were washed and resuspended with 200 mL of a precooled extraction buffer. The supernatants were divided into two parts, whereby one was used to determine the total amount of NAD^+^/NADH, and the other was used to determine the total amount of NADH only. All steps were performed according to the NAD^+^/NADH test kit of WST-8 (Beyotime).

#### 4.10.8. Bacterial Respiration

Strains that were grown overnight before and after induction were diluted at 1:100 in 1 mL of fresh MHB and grown to the exponential stage for 4 h, after which the bacterial cells were resuspended in M9 broth (OD_600_ = 0.5). A ciprofloxacin solution with a final concentration of 20 × MIC and iodonitrotetrazolium chloride (INT) (Sigma) with a final concentration of 2 mM were added to the bacterial cultures. The bacteria were evenly distributed in 96-well plates, and the absorbance of the bacteria solutions at 485 nm was measured for 60 min using a microplate reader. At the same time, the bacterial solutions were poured into a 96-well plate, and azurol was added to the cultures at a final concentration of 0.01 mg/mL. Ciprofloxacin or its combination with exogenous metabolites was then added at 20 × MIC. Fluorescence units were measured at an excitation wavelength of 550 nm and an emission wavelength of 590 nm for 30 min.

#### 4.10.9. Total ROS Measurement and Determination of T-SOD Activity 

The reactive oxygen species (ROS) and total superoxide dismutase (T-SOD) activities in *E. coli* O157:H7, CA-*E. coli* O157:H7, *E. coli* B2, and CA-*E. coli* B2 were determined using the Reactive Oxygen Species Assay Kit (Beyotime) and the Total Superoxide Dismutase Assay Kit with WST-8 (Beyotime). The intracellular levels of ROS and T-SOD after the addition of xanthine were determined using the same method.

#### 4.10.10. Effect of Bactericidal Activity of Antibiotics on Changes in Exogenous Metabolites

To study the effect of amino acids on citric-acid-induced antibiotic tolerance, *E. coli* O157:H7 cells cultured for 6 h were harvested, washed, and resuspended in MHB and then treated with ciprofloxacin (20 × MIC) and 6 different amino acids (10 mM) for a further 6 h. Then, the remaining CFU were counted, and the corresponding bacterial yield was calculated to determine the bactericidal activity of the antibiotics.

### 4.11. Animal Studies

Female BALB/c mice aged 6–8 weeks (18–20 g) were obtained from the Animal Experiment Center of Jilin University (Jilin, China). All experiments were conducted under the Regulations of the Jilin Laboratory Animal Management Committee of Jilin Province. The license number of the experimental animals was JLAU20210423001, which has certification from the Jilin Science and Technology Association.

#### 4.11.1. Treatment Tests in Mice for Acute Toxicity

Female BALB/c mice (n = 20 per group) were given a 1.0 × 10^8^ CFU suspension of *E. coli* B2 and CA-*E. coli* B2. After 2 h of infection, all infected mice were randomly divided into control and antibiotic-treated groups (n = 10 biologically independent animals in each group). A single dose of PBS and ciprofloxacin (50 mg/kg) was injected intramuscularly, respectively. The survival rates of the mice were recorded for 7 days.

#### 4.11.2. Bacterial Enteritis Infection Model in Mice

Female BALB/c mice (n = 20 per group) were given sterile PBS or 20 g/kg of citric acid for 5 days, followed by a 2.0 × 10^8^ CFU *E. coli* B2 or CA-*E. coli* B2 suspension. All infected mice were randomly divided into a control group and an antibiotic-treated group (n = 10 biologically independent animals in each group) 2 hours after infection and a single dose of PBS and ciprofloxacin (50 mg/kg) was injected intramuscularly, respectively. The survival rates of the mice were recorded for 7 days.

#### 4.11.3. Galleria mellonella Infection Model

*Galleria mellonella* larvae (Tianjin Huiyude Biotechnology Co., Ltd., Tianjin, China) were randomly divided into 5 groups (n = 10 biologically independent animals in each group). An *E. coli* O157:H7 or CA-*E. coli* O157:H7 suspension (10 μL per larva; 1.0 × 10^6^ total colony count; 5 groups) was injected into the left posterior of the moths 1 hour after infection, and PBS, ciprofloxacin (50 mg/kg), or a combination of ciprofloxacin and succinic acid or xanthine (50 + 20 mg/kg) were injected into the left posterior of the gastropods. The survival rates of the *Galleria mellonella* larvae were calculated daily for the next five days.

#### 4.11.4. Acute Toxicity Rescue Test in Mice

Female BALB/c mice (n = 20 per group) were given sterile PBS or 20 g/kg citric acid for 5 days, followed by a 2.0 × 108 CFU *E. coli* B2 or CA-*E. coli* B2 suspension. Two hours after infection, all infected mice were randomly divided into a control group, an antibiotic-treated group, and an antibiotic combined with metabolites-treated group (n = ten biologically independent animals in each group). PBS, ciprofloxacin (50 mg/kg), or a combination of ciprofloxacin and succinic acid or xanthine oxidase (50 + 20 mg/kg) were injected intramuscularly. The survival of the mice was recorded for 7 days.

### 4.12. Statistical Analyses

Statistical analysis was performed with GraphPad Prism Version 8.3.0. All data from at least three biological replicates are presented as means ± SD. Unless otherwise stated, an unpaired *t*-test between 2 groups or a one-way ANOVA between multiple groups was used to calculate *p*-values (ns, not significant; * *p* < 0.05, ** *p* < 0.01, *** *p* < 0.001, and **** *p* < 0.0001).

### 4.13. Data Availability

The transcriptome data on the bacteria of this study have been uploaded to the NCBI GenBank, Gene Expression Omnibus (GEO) database under accession number GSE226409.

## Data Availability

The transcriptome data on the bacteria in this study have been uploaded to the NCBI GenBank, Gene Expression Omnibus (GEO) database under accession number GSE226409. The dataset generated and analyzed in this study can be obtained from the corresponding authors upon reasonable request.

## Supplementary Materials

The following supporting information can be downloaded at https://www.mdpi.com/article/10.3390/ijms24109089/s1.
