# Supplementary material for: Citric Acid Confers Broad Antibiotic Tolerance through Alteration of Bacterial Metabolism and Oxidative Stress
Source: Int J Mol Sci. 2023 May 22;24(10):9089. doi: 10.3390/ijms24109089 (PMC10219361; doi:10.3390/ijms24109089)
Supplement: Supplementary file 1 [file ijms-24-09089-s001.zip › ijms-2316860-supplementary.pdf]

Supplementary Materials:

Table S1. MIC analysis of citric acid against five representative strains.

Pathogens	MIC (μg/mL)
E. coli ATCC 25922	>512
E. coli B2	>512
E. coli O157:H7	>512
S. aureus ATCC 29213	>512
MRSA T144	>512

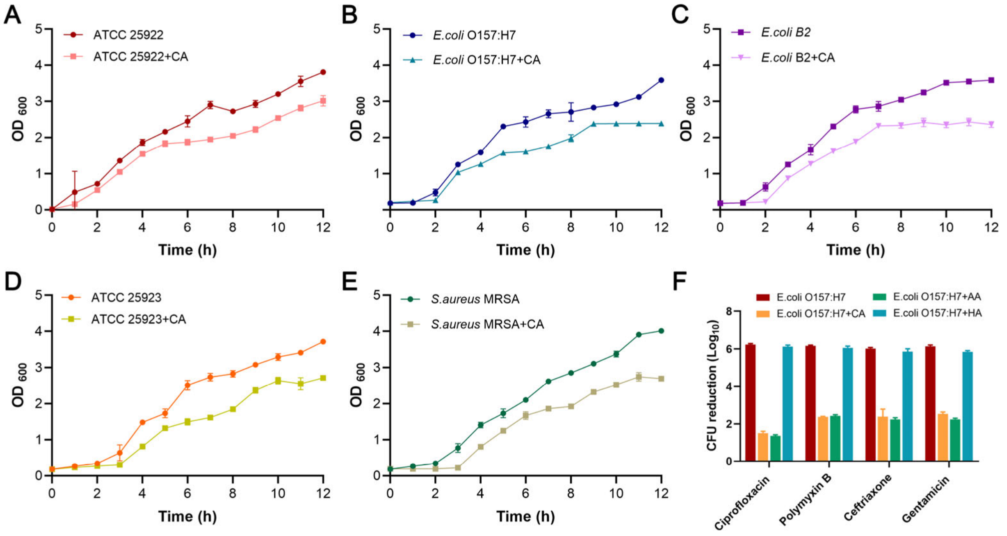

Figure S1 Growth curves of five representative strains grown in the presence of citric acid (final concentration 2%) for 12 hours. The optical density (OD_{600}) of citric acid and (A) *E.coli* ATCC 25922, (B) *E.coli* O157:H7, (C) *E.coli* B2, (D) *S.aureus* ATCC 25923, (E) MRSA T144 were monitored within 12 hours.（F）：Effect of adding different acidifiers on bactericidal activity of bactericidal antibiotics. CA: Citric acid, AA: Acetic acid, HA: Hydrochloric acid. After adding three acidifiers, the pH of the culture medium is about 2.2. Three biological replicates were used for the experiment.

Table S2 Co-culture of five representative strains with citric acid had no effect on MIC value of drugs

μg/mL

Antibiotic	Targets	*E. coli* ATCC 25922		*E. coli* O157:H7		*E. coli* B2		*S. aureus* ATCC 29213		MRSA T144	
		MIC [a]	MIC [b]	MIC [a]	MIC [b]	MIC [a]	MIC [b]	MIC [a]	MIC [b]	MIC [a]	MIC [b]
CIP	DNA synthesis	0.03	0.03	<0.03	<0.03	32	32	0.5	0.5	8	16
CRO	Cell wall	0.03	0.03	<0.03	<0.03	256	256	4	4	>512	>512
PB	Cell membrane	0.5	1	0.5	0.5	2	2	-	-	-	-
GEN	Protein synthesis	0.5	0.5	0.5	0.5	64	64	0.5	0.5	128	128
KAN	Protein synthesis	2	2	2	2	512	512	2	2	512	512
TIG	Protein synthesis	0.125	0.125	0.03	0.03	0.5	0.5	0.125	0.125	0.125	0.125
VAN	Cell wall	-	-	-	-	-	-	0.5	1	0.5	0.5

[a] Initial MIC of representative strains against antibiotics;

[b] MIC of representative strains after co-culturing with citric acid (final concentration 2%) for 6 h.

Abbreviations: CIP, Ciprofloxacin; CRO, Cefatriaxone; PB, Polymyxin B; GEN, Gentamicin; KAN, Kanamycin; TIG, Tigecycline; VAN, Vancomycin.

Table S3 MIC analysis of five antibiotics before and after citric acid induced antibiotic tolerance in *E.coli* B2.

Antibiotic	MIC (μg/mL)[a]	MIC (μg/mL)[b]
Cefatriaxone	256	256
Ciprofloxacin	32	32
Polymyxin B	2	2
Gentamicin	64	64
Kanamycin	512	512

[a] Initial MIC of *E.coli* B2 against antibiotics;

[b] MIC of CA-*E. coli* B2 induced by citric acid.

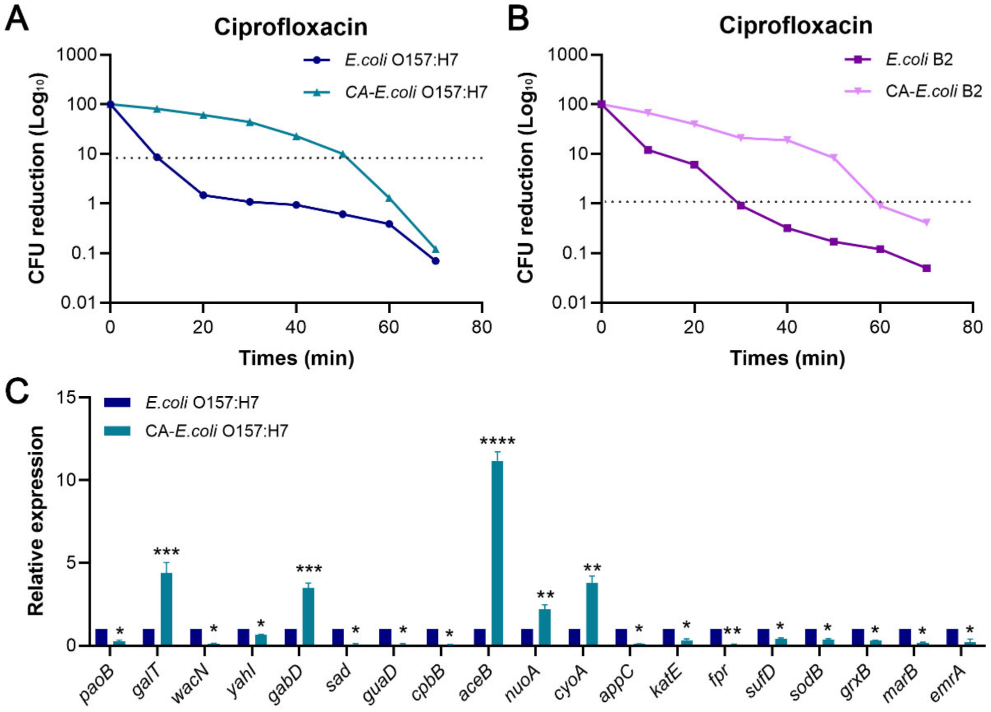

Figure S2: Growth status and mRNA expression of antibiotic tolerance strains induced by citric acid. (A and B): Determination of the growth rate of E. coli and CA-E. coli strains after stable inheritance of citric acid-induced antibiotic tolerance. Observe the absorbance value at OD_{600} nm of different strains within 12 hours. Three biological replicates are used. (C): The transcriptome sequencing results of the strains before and after citric acid induction were verified by RT-qPCR. All data are presented as mean ± SD and the significances determined by unpaired t test. All data from biological experiments performed in triplicate are presented as means ± SD. *$P < 0.05$, **$P < 0.01$, ***$P < 0.001$, ****$P < 0.0001$.

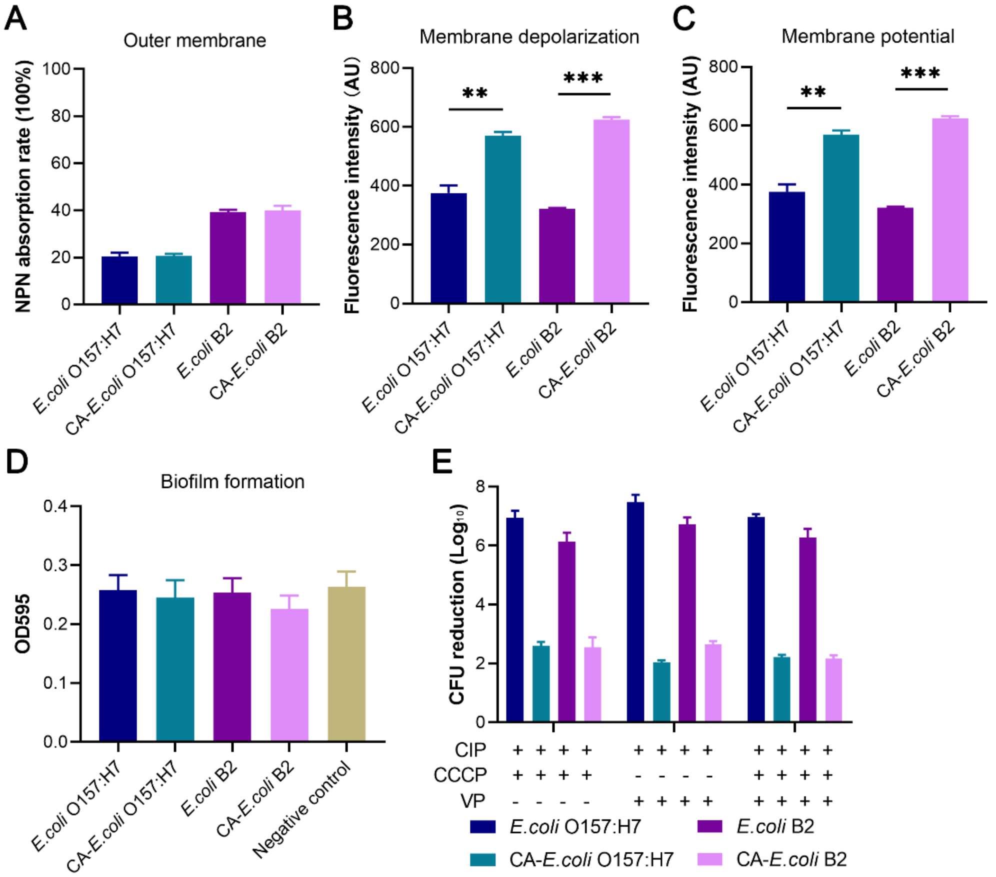

Figure S3: **Effects of citric acid-induced antibiotic tolerance on outer membrane integrity, membrane permeability, membrane potential changes and biofilm formation of *E. coli* strain.** (A and D): The outer membrane integrity and biofilm formation of the *CA-E.coli* strain did not change compared to the *E.coli* strain. (B and C): However, in the CA-*E. coli* strain, membrane depolarization and membrane potential dissipation occurred. (E): CCCP (efflux pump inhibitor, 100 μM) and VP (calcium channel inhibitor, 100 μM) alone or in combination did not affect the bactericidal activity of ciprofloxacin against CA-*E. coli*. All data from biological experiments performed in triplicate are presented as means ± SD. $**P < 0.01$, $***P < 0.001$.

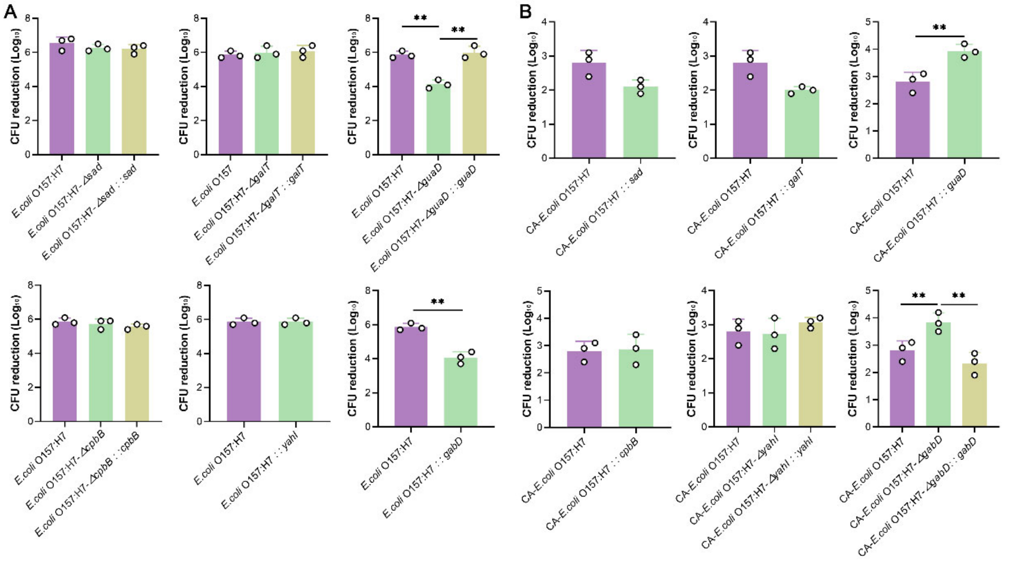

Figure S4: **Effects of *guaD* gene and *gabD* gene on antibiotic tolerance in *E.coli*.** A: Deletion and deletion-complemented of *guaD* gene and overexpression of *gabD* gene were constructed in *E.coli* O157:H7 strain. It was found that deletion of the *guaD* gene and overexpression of the *gabD* gene could improve antibiotic tolerance to some extent. B: Overexpression of the *guaD* gene , deletion and deletion-complemented of the *gabD* gene were constructed in CA-*E.coli* O157:H7 strain. It was also found that overexpression of the *guaD* gene and deletion of the *gabD* gene could restore the tolerance of the strain to ciprofloxacin to a certain extent. All data from biological experiments performed in triplicate are presented as means ± SD. $^{**}P < 0.01$.

Table S4 Strains used in this study.

Strains	Source/Reference
**Gram-negative bacteria**	
E. coli ATCC 25922	ATCC
E. coli O157:H7	In this study
E. coli B2 (*mcr-1* + *bla*$_{NDM-5}$)	In this study
E. coli O157:H7 Δ*galT*	In this study
E. coli O157:H7 Δ*galT :: galT*	In this study
E. coli O157:H7 Δ*wcaN*	In this study
E. coli O157:H7 Δ*wcaN :: wcaN*	In this study
E. coli O157:H7 Δ*sad*	In this study
E. coli O157:H7 Δ*sad :: sad*	In this study
E. coli O157:H7 Δ*guaD*	In this study
E. coli O157:H7 Δ*guaD :: guaD*	In this study
E. coli O157:H7 Δ*cpbB*	In this study
E. coli O157:H7 Δ*cpbB :: cpbB*	In this study
E. coli O157:H7 *:: yahI*	In this study
E. coli O157:H7 *:: gabD*	In this study
**Gram-positive bacteria**	
S. aureus ATCC 29213	ATCC
MRSA T144	In this study

ATCC, American Type Culture Collection.

Table S5 RT-qPCR primers used in this study.

Genes	Sequence (5'→3')	Product (bp)
paoB	AAGGTGTTTTCAGCGGTGGGATG<br>CTGGGCGGAGTAGCACATAAGC	122
yahI	CGGCATCGGCTATCTGATCCAAC<br>TCCACTTCCACCTGAGTCACCAC	96
wcaN	GCAGCCACAATCTCGTCAACAATG<br>GCAGTTATTCCTGTAGCGGGTCTC	125
sad	ATTCAGCCAGCCATATACGCCTTG<br>TTCCCATCATTCTTGCAGGTAACGG	128
galT	AGCTGCCAGTGTTGATTCTCTTCG<br>GACAACCTCTTCCAGTGCTCCTTC	86
cpdB	AGCACGAAACCACAGTCACTCATC<br>CCTGACGGTTGATCTTCGCT	102
guaD	TGCCTGGGTGAAATCGCTTTATCC<br>AGATGGACGCAGTGAGCAAAGAC	105
fixA	GTATGGTCGGCGGCGGATATTG<br>GCAAATGCGGCGATCTGTTCTTC	134
fixX	CAAGAAGCAGGATGACGGCAGTG<br>CGTAACGGAACTCCACACCAAAGG	140
folA	TGACGCATATCGACGCAGAAGTG<br>CCGCCGCTCCAGAATCTCAAAG	143
polB	AGTTCACCCGCCATCAGTTTGTC<br>GCCCAGCAGTTTCAGCAGGAG	101
copA	TACGCTCCGCCAGGTTTACCC<br>ATGACGATGACAGCCAGCAGTTG	126
degP	CAACGGCGGCTGAGACTTCTTC<br>GCTGACCACTGAAGGCATCACC	92
rclA	TCAATGCCCATGTGGAGCGAATC<br>CTGGATGTAACGAAGCGGTAGCC	132
csgA	CGGTGGTGGCGGTAATAATAGCG<br>GTCAAGTCAGAGTTACGGGCATCAG	111
ydcL	CACCGAAATGAAAGAAGCGATAGCG<br>GCCCCACGGAAAATCAGACTACG	81
aceF	AGAACGAAGAAGCGGCGAAACG<br>AGCGAACTATTGAAGCGAGGCATC	112
emrA	CGTGCGTCTTCCTCGGTAATGTAG<br>CACCTCTTCTATCGGCGTTGTCAC	117
16S rRNA	AACTGGAGGAAGGTGGGGATGAC<br>CGGACTACGACGCACTTTATGAGG	135
